# A phase II trial of bryostatin-1 administered by weekly 24-hour infusion in recurrent epithelial ovarian carcinoma

**DOI:** 10.1038/sj.bjc.6601285

**Published:** 2003-09-30

**Authors:** A R Clamp, F H Blackhall, P Vasey, M Soukop, R Coleman, G Halbert, L Robson, G C Jayson

**Affiliations:** 1 Cancer Research UK Department of Medical Oncology, Christie Hospital NHS Trust, Wilmslow Road, Withington, Manchester, M20 4BX, UK; 2 Clinical Trials Unit, Cancer Research UK Department of Medical Oncology, Beatson Oncology Centre, Western Infirmary, Glasgow G11 6NT, UK; 3 Department of Medical Oncology, Royal Infirmary, 84 Castle Street, Glasgow, G4 0SF, UK; 4 Department of Clinical Oncology, Weston Park Hospital NHS Trust, Whitham Road, Sheffield S10 0SJ, UK; 5 Cancer Research UK Formulation Unit, Department of Pharmaceutical Science, University of Strathclyde, Royal College Building, 204 George Street, Glasgow G1 1XW, UK; 6 Drug Development Office, Cancer Research UK

**Keywords:** bryostatin-1, ovarian cancer, protein kinase C, clinical trial

## Abstract

Bryostatin-1 is a macrocyclic lactone whose main mechanism of action is protein kinase C modulation. We investigated its activity as a weekly 24-h infusion in recurrent ovarian carcinoma. In all, 17 patients were recruited and 11 had chemotherapy-resistant disease as defined by disease progression within 4 months of last cytotoxic therapy. All were evaluable for toxicity and 14 for response. There were no disease responses and the main toxicity was myalgia.

Although advanced epithelial ovarian cancer is sensitive to platinum-containing chemotherapy at first presentation, the majority of patients experience disease relapse and their management at this stage is often difficult. While rechallenge with platinum is appropriate for patients with at least a 6-month disease-free interval (Markman *et al*, 1991), those who progress sooner require treatment with newer agents. The response rates to these, however, are disappointing and therefore a clear need exists for the development of novel approaches to the management of recurrent ovarian carcinoma.

Bryostatin-1 is a naturally occurring macrocyclic lactone with antineoplastic activity derived from the marine bryozoan, *Bugula neritina* (Pettit *et al*, 1982). *In vitro* evidence indicates that its main mechanism of action is the modulation of protein kinase C activity. How this is antineoplastic is less clear, but may involve apoptosis induction or immunomodulatory effects (Clamp and Jayson, 2002).

During Phase I evaluation of bryostatin-1, myalgia was shown to be the main dose-limiting toxicity (Philip *et al*, 1993; Prendiville *et al*, 1993; Varterasian *et al*, 1998), although the mechanism for this is not fully understood. A maximum tolerated dose of 25 *μ*g m^−2^ week^−1^ was identified when a weekly 24-h infusion was used (Jayson *et al*, 1995). Of note, one partial and one minor response occurred in two patients with relapsed ovarian cancer in the trial and so we undertook a Phase II study with this regimen to evaluate further the efficacy of bryostatin-1 in this group of patients.

## MATERIALS AND METHODS

### Trial design

This study was a multicentre open label nonrandomised Phase II trial. The predefined endpoints were response rate and progression-free interval. To ensure a low probability (*P*<0.05) of erroneously rejecting a treatment that is active in 20% of patients, a minimum of 14 subjects were planned to be treated (Gehan, 1961).

The study was approved by the Cancer Research Campaign Phase I/II Committee and Central Independent Review Board (CIRB), the National Cancer Institute and Local Regional Ethics Committees. Written informed consent was obtained from all patients.

### Patients

Patients eligible for inclusion were at least 18 years old, with histologically proven epithelial ovarian cancer that had progressed during or after at least one platinum-containing chemotherapy regimen. The progression-free interval following last chemotherapy treatment was not a defined selection criterion. All patients had a life expectancy of ⩾12 weeks and a WHO performance status of 0–2. They had radiologically measurable disease ⩾2 cm in two dimensions.

Adequate biochemical and haematological functions were documented within 1 week of trial entry. Patients were excluded if they had received more than two prior multidrug regimens for ovarian cancer and other standard Phase II exclusion criteria applied. Systemic steroid administration was contraindicated while patients were receiving bryostatin-1.

### Formulation, dose and administration

Bryostatin-1 (US National Cancer Institute, Arizona State University, USA) was stored at 4°C in flint vials containing 0.1 mg of lyophilised bryostatin-1. Prior to administration, it was dissolved in 1 ml of PET (60% polyethylene glycol, 30% ethanol, 10% Tween 80) diluent and then diluted with 9 ml of 0.9% sodium chloride. The prescribed volume was then drawn up into a polypropylene syringe and co-infused intravenously over 24 h with 2 l of 0.9% sodium chloride. Bryostatin-1 was administered weekly at a dose of 25 *μ*g m^−2^ for a planned eight doses.

In the presence of Grade 3 or 4 myalgia and/or headache, treatment was delayed for 1 week. If Grade 3 toxicity persisted, the patient was withdrawn from the trial. If myalgia/headache had improved to Grade 2 or better, treatment was recommenced at a 25% dose reduction (19 *μ*g m^−2^). If ⩾Grade 3 toxicity recurred, treatment was discontinued.

If any other Grade 3/4 toxicity (or Grade 1 thrombocytopenia) occurred, treatment was delayed for 1 week and was then restarted at 19 *μ*g m^−2^ if recovery had occurred to Grade ⩽1 (except for thrombocytopenia (Grade 0) and neutropenia (Grade 2)). Recurrent toxicity led to treatment withdrawal. If Grade ⩾2 phlebitis occurred, treatment was delayed for 1 week and then recommenced at 25 *μ*g m^−2^. If phlebitis recurred, a 25% dose reduction was instituted.

### Monitoring of toxicity and response

Patients were reviewed by a physician weekly, prior to the administration of bryostatin-1. Included in this review were the patient's WHO performance status and the documentation of any new symptoms and adverse events according to the NCI-CTC criteria (version 2.0). A full blood count and serum biochemistry were performed weekly.

Evaluable and measurable disease sites were assessed after 8 weeks of treatment by the same imaging modality employed prior to study entry. Serum CA-125 levels were determined weekly. Patients with progressive disease were withdrawn from the study. Patients with clear clinical progression were deemed evaluable for response, provided that they had received at least four infusions of bryostatin-1. Those patients who had not received four infusions were replaced.

## RESULTS

### Patient demographics

In all, 17 patients (median age 60 years, range 43–71) were recruited. All patients had undergone surgery and platinum-based chemotherapy after diagnosis. A total of 11 of 16 evaluable patients had responded to first-line therapy, while two had progressive disease. 13 patients subsequently received second-line cytotoxics prior to trial entry. At recruitment all patients had documented disease progression, 11 had chemotherapy-resistant and nine had platinum-resistant disease as defined by progressive disease on treatment or disease relapse within 4 months of discontinuing therapy. Nine patients had WHO performance status 0, 6 PS1 and 2 PS2 at entry.

### Bryostatin-1 administration and response

In total, 95 doses of bryostatin-1 were given to 17 patients with a median of six doses each (range 2–9). Four were administered at 19 *μ*g m^−2^ due to toxicity. Eight dose delays of 1 week or more occurred in eight patients. Two were due to toxicity (one myalgia, one lethargy), three due to concurrent medical problems, one at patient request due to Grade 2 myalgia and two due to administrative constraints.

Three patients were considered nonevaluable due to marked clinical deterioration prior to completing 4 weeks of bryostatin-1 therapy. In all three cases, this was due to rapidly progressive disease. In all, 14 patients were evaluable. No radiological or CA-125 responses were noted on therapy. Six patients had early disease progression as defined by clear clinical and/or radiological evidence of progression prior to the planned assessment after eight doses of bryostatin-1. Four patients had progressive disease and four patients showed disease stabilisation after eight doses. In all patients with stable disease, treatment was discontinued at the patients’ request due to the deteriorating quality of life associated with weekly hospital attendances and the side effects of bryostatin-1. Two of these patients commenced other cytotoxic treatment immediately on withdrawal. In addition, one patient showed disease stabilisation off antineoplastic treatment for 12 months. In the last patient with stable disease, a 94% fall in serum CA-125 was documented 13 weeks after discontinuing bryostatin-1 and disease regression was confirmed radiologically.

### Toxicity

Toxicities attributed to bryostatin-1 are summarised in Table 1

**Table 1:** Toxicity probably/definitely attributable to bryostatin-1 (25 *μ*g m^−2^)

	**NCI-CTC v2.0-grading**		
**Toxicity**	**2**	**3**	**4**
Myalgia	4	4	—
Fatigue	1	2	—
Vomiting	—	1	—
Lymphocytopenia	6	—	—
Dermatology-other	2	—	—
Haemoglobin	2	—	—
Phlebitis	2	—	—
Anorexia	2	—	—
Constipation	2	—	—
Nausea	1	—	—
Diarrhoea	1	—	—
Muscle weakness	1	—	—
Ocular-other	1	—	—
Elevated *γ*-GT	1	—	—
Arthralgia	1	—	—
Headache	1	—	—
Injection site reaction	1	—	—
Stomatitis	1	—	—

Number of patients with Grade 2 toxicities and above are listed. Only the worst grade of toxicity noted in each patient is included.

. Eight patients experienced Grade II or III myalgia. This usually became evident after the second bryostatin-1 infusion and its intensity and duration increased as the treatment continued. Three patients who experienced Grade 3 myalgia withdrew from the trial at their own request. One patient was withdrawn due to Grade 3 lethargy. Notably, the performance status of 13 patients deteriorated during therapy.

## DISCUSSION

### Efficacy

Although activity in epithelial ovarian cancer was documented in a Phase I study of bryostatin-1 administered as a weekly 24-h infusion (Jayson *et al*, 1995), no disease responses were seen in this Phase II study. This is in keeping with the disappointing results observed in single-agent Phase II studies performed in other disease groups using a variety of administration regimens (summarised in Clamp and Jayson, 2002).

It should be noted, however, that although treatment free interval was not a selection criterion for trial entry, the patient population treated in this study primarily had chemotherapy-resistant disease. This group is known to have response rates of 0–27% to novel agents tested in the Phase II setting (Latorre *et al*, 2002). Using the statistical principles applied (Gehan, 1961), we can only formally exclude activity at the 20% level and so cannot discount equivalent activity with some cytotoxic agents in clinical usage.

The demonstration of prolonged stable disease in one patient is consistent with previous reports with bryostatin-1, particularly in hypernephroma (Pagliaro *et al*, 2000), although these patients continued on therapy until progression. The observation of delayed disease regression in one patient is intriguing, but difficult to attribute to bryostatin-1 as initial evidence of response was only noted 3 months after therapy was discontinued.

### Toxicity

Bryostatin-1 caused significant myalgia, with 48% of patients experiencing at least Grade 2 myalgia on treatment. The short duration of therapy, the rapid onset of myalgia and its increasing duration and intensity with cumulative doses all indicate that this side effect is likely to be a significant impediment to the further clinical development of this agent. This is further reinforced by the fact that three of the four patients in this study experiencing Grade 3 myalgia withdrew from treatment at their own request despite formal documentation of stable disease.

### Combination regimens

Given its unique side effect profile, it may be that the future clinical niche for bryostatin-1 in ovarian cancer will be as part of combination regimens with conventional cytotoxic agents. Indeed, preclinical data demonstrate synergy with both cisplatin and paclitaxel (Basu and Lazo, 1992; Koutcher *et al*, 2000) and the safety of cisplatin/bryostatin-1 combinations has already been demonstrated (Rosenthal *et al*, 1999). It is particularly intriguing that encouraging Phase II activity with the combination of cisplatin and tamoxifen has been reported (Benedetti Panici *et al*, 2001), as one of tamoxifen's possible mechanisms of action is the modulation of PKC activity, suggesting that it could mimic bryostatin-1 (Schwartz *et al*, 2002). The combination of bryostatin-1 and paclitaxel, however, may be hampered by severe myalgias as reported in one study in oesophageal carcinoma (Kortmansky *et al*, 2002).

In summary, the results of this trial indicate that bryostatin-1 administered alone at a dose of 25 *μ*g m^−2^ as a weekly 24-h infusion has no role in the management of relapsed chemotherapy-resistant epithelial ovarian cancer. Its future utility will depend on the successful evaluation of combination regimens with other cytotoxic agents with which it has no overlapping toxicities.
